# “I don’t need my kid to be high”: prioritizing harm reduction when using cannabis during pregnancy

**DOI:** 10.1186/s12954-024-01046-2

**Published:** 2024-09-09

**Authors:** Erin E. Gould, Siddhi S. Ganesh, Rachel Carmen Ceasar

**Affiliations:** Department of Population and Public Health Sciences, Keck School of Medicine, University of Southern California, 1845 North Soto Street, Los Angeles, CA 90032 USA

**Keywords:** Cannabis, Harm reduction, Risk, Pregnancy, Stigma, Intervention, Safety

## Abstract

**Background:**

Cannabis is the most common illicit substance used in pregnancy. As use continues to increase, understanding peoples’ behaviors surrounding cannabis use during pregnancy is needed to improve maternal and child health outcomes. The aim of this study was to better understand pregnant individuals' perceptions and knowledge of cannabis use and use patterns as well as the social and environmental factors that may influence their use.

**Methods:**

We conducted interviews with 19 participants between December 2022 and March 2023. Individuals self-identified as BIPOC (Black, Indigenous, People of Color), were over 21 years of age, spoke English or Spanish, resided in California, and had used cannabis during pregnancy in the last 0–2 years. Using qualitative, constructivist grounded theory methods, we analyzed the contexts that contributed to participants’ lived experiences surrounding cannabis use behaviors during pregnancy.

**Results:**

Participants reported making conscious decisions to responsibly manage their cannabis use during pregnancy to minimize potential harm to the fetus. Participants prioritized making what they perceived to be safer adjustments to their use of cannabis: (1) changing the amount of cannabis used, (2) changing the types of cannabis products used, and (3) changing sources of cannabis procurement.

**Discussion:**

Our findings show that pregnant individuals are seeking information about safe cannabis use beyond medical supervision and are open to altering their cannabis consumption patterns. However, they are unable to find trustworthy and evidence-based harm reduction practices which can be implemented to mitigate harm to their unborn children. A harm reduction approach is needed in the field of maternal cannabis use to promote positive maternal and fetal health outcomes.

**Conclusions:**

More data is needed on comprehensive harm reduction approaches to cannabis use during pregnancy. This requires implementation of education on these topics in healthcare settings presented by prenatal care clinicians.

**Supplementary Information:**

The online version contains supplementary material available at 10.1186/s12954-024-01046-2.

## Introduction

Though federally illegal in the United States, cannabis legality varies by state and can be accessed in most of the country [1, 2]. It is medically legal in 38 states and recreationally legal in 24 states 1, 2. Following legalization, rates of cannabis use during pregnancy may increase [3–5]. Cannabis is the most common illicit substance used during pregnancy with use rates ranging from 2 to 36% and increasing in past years [3, 6–8]. The prevalence of substance use in pregnancy, including tobacco, alcohol, and illicit drugs such as cannabis, is estimated to be between 8 and 11%. 6 One study reported that between 2002 and 2020, the prevalence of cannabis use in pregnancy increased, alcohol use decreased, and cannabis-alcohol co-use slightly increased [9]. Cannabis use during pregnancy is widely discouraged by organizations such as The American College of Obstetricians and Gynecologists (ACOG) and The American Academy of Pediatrics (AAP) due to concerns over neonatal health impacts [10]. Cannabis use in pregnancy is associated with giving birth to infants with low birth weight and small gestational size, but there is low evidence for additional effects [11]. The existing data on maternal cannabis use identifies that more research is needed on cannabis use in the perinatal period to better predict outcomes and possible interventions [5, 12–14].

Despite the potential negative fetal implications, there are a myriad of reasons pregnant individuals choose to use cannabis, such as for symptom management of nausea or vomiting [13, 15–18]. Pregnant individuals report using cannabis within a healthcare landscape offering few alternative options for symptom management [15]. While there are some pharmaceutical interventions for nausea and vomiting which are safe for the fetus [19, 20], pregnant individuals report that these medications are ineffective or produce intolerable side effects leading to the feeling that care is fetus-centric rather than incorporating their immediate needs. [15, 21] Patients report low-quality or absent counselling on maternal cannabis use from clinicians, with conversations centering legal action rather than well-being. [14, 15, 21].

Physicians report reluctance to counsel pregnant patients about cannabis due to unclear information and concerns regarding maintaining a therapeutic alliance with patients, often leaving patients to navigate this situation on their own [12, 15]. Universal substance use screening is recommended by ACOG in the perinatal period, but this practice is often higher for those of minoritized identities [22] and in different care settings [12, 15]. Despite the increasing legality and prevalence of cannabis use, disclosing cannabis use in pregnancy often results in stigma and legal action such as Child Protective Services (CPS) involvement [5, 21] which is disproportionately higher for patients of minoritized identities [5, 7, 23].

Given the stigmatization alongside the paucity of dialogue surrounding cannabis use during pregnancy in healthcare spaces, pregnant individuals often feel forced into navigating cannabis use on their own without medical supervision 15. Scant reliable data on harm reduction measures regarding cannabis use in pregnancy exists [24–26], despite pregnant individuals’ desire for it [27]. Decisions regarding whether to use cannabis during pregnancy are complex, involving an analysis of risk versus benefit as individuals navigate their own health alongside their unborn child’s [28]. That individuals are turning to cannabis as a perceived viable option to manage pregnancy symptoms is widely seen [28, 29]. Less is known about the specific methods by which pregnant individuals manage and/or change their cannabis use throughout the prenatal period. The aim of this study was to better understand pregnant individuals' perceptions and knowledge of cannabis use and use patterns as well as the social and environmental factors that may influence their use.

## Methods

### Study design: constructivist grounded theory

This research consisted of qualitative interviews with 19 people (n = 19) who use cannabis during pregnancy. Our research team consisted of undergraduate students, master's students, doctoral students (SSG), research staff (EEG), and a faculty member (RCC) acting as the Principal Investigator of the study. We reviewed SRQR (standards for reporting qualitative research), a validated 21-item checklist for qualitative reporting, throughout the study to document important aspects of our research team, methodology, findings, and analysis (Appendix 1: SRQR checklist) [30, 31]. We conducted a phenomenological study using constructivist grounded theory that aimed to understand the perspectives of individuals who used cannabis during pregnancy [32]

### Selection of participants: sampling and recruitment strategies

We recruited via online social media advertisements which directed potential participants to a HIPAA-compliant REDCap survey to confirm eligibility and schedule an orientation session, which included informed consent, and interview. To be eligible, participants self-identified as BIPOC (Black, Indigenous, People of Color), were over 21 years of age, spoke English/ Spanish, resided in California, and had used cannabis while pregnant in the last 0–2 years.

Study participants were only eligible if they were at least 21 years of age due to laws and regulations in the state of California regarding the use, possession, sharing, and growing of cannabis. While this study focused on women who use cannabis during pregnancy, the study of fetuses, neonates, or children was beyond the scope of this research. Because we are interested in experiences with cannabis during pregnancy, only pregnant or postpartum women were part of this study. To better understand the unique environment and effects of cannabis-related state laws that have expanded legalization, accessibility, and acceptability, all study participants needed to have lived in California at the time of recruitment.

Minoritized individuals and their communities are disproportionately harmed by screening and reporting policies surrounding perinatal substance use, discrimination, and reproductive health inequities. This research sought to collect data on an understudied group which is most vulnerable to the negative consequences surrounding expanding legalization of cannabis.

It was a novel examination of contextual factors as drivers of risk influencing maternal health disparities from a diverse study population (i.e., BIPOC individuals) using a multidimensional framework. Selection of this sample was necessary for generating data on the perceptions of BIPOC individuals who use cannabis during pregnancy and how these perceptions may worsen maternal health disparities.

We used a theoretical sampling strategy, wherein recruitment continued until theoretical saturation was achieved, to arrive at 19 participants. Theoretical sampling is a grounded theory data collection methodology in which sampling is guided by emerging theory [33, 34]. As we underwent data collection efforts, new theoretical categories emerged and were built upon in subsequent interviews until no new ones occurred, thus theoretical saturation was achieved, and data collection was complete.

### Data collection: semi-structured interviews

The 60-min interview sessions occurred remotely via HIPAA-compliant Zoom™ video calls between December 2022 and March 2023. We used a semi-structured interview guide which drew upon existing qualitative and quantitative literature (Appendix 2: Interview Guide) [14, 35–38]. Questions were developed using the National Institute on Minority Health and Health Disparities framework (NIMHD) wherein questions were tied to each level of influence (i.e., individual, interpersonal, community, and societal) and domain of influence (i.e., biological, behavioral, physical/ built environment, and healthcare system) as they relate to health outcomes [38]. The NIMHD Research Framework facilitates understanding the importance of contextual factors as drivers of risk that may influence substance use among marginalized groups because it: (1) offers a systematic approach to identify the environmental, social, political, and cultural influences on the health of individuals that are often overlooked in epidemiological survey research (e.g., fear of consequences for cannabis use by individuals of minoritized identities), and (2) locates influences that may be especially relevant to understanding the health and well-being of BIPOC pregnant individuals, for whom social determinants may be salient but which are often not considered by conventional research because they reside outside of the normal experience of non-BIPOC groups (e.g., historical trauma of losing parental rights for use of substances, avoidance of prenatal care for fear of punitive action). This framework was applied to the current study to understand how expanding legalization and social acceptability and accessibility of cannabis may worsen existing maternal health disparities for individuals in marginalized groups. This was further developed using questions posed in other qualitative studies on maternal cannabis use (i.e., *“Where do women get information about marijuana use during pregnancy?”)* to adapt questions to our specific sample [35].

The interview guide questions (i.e., *How did you learn about cannabis for pregnancy? What information did you wish you had?*) were piloted and refined within the research team. We met weekly to discuss data collection and participant recruitment. During these meetings we iteratively revised the interview guide as the interviews progressed. This was to refine questions and pursue areas identified as theoretically relevant. This method of iterative revision is aimed at generating richer responses from participants [34, 39].

Each interview was conducted by 1–2 research team members, with one individual leading the discussion and the other co-leading and taking analytical notes to inform analysis. We followed up on questions (probes) with open-ended inquiries about topics introduced by the participants (i.e., *What kind of feedback did you receive for disclosing cannabis use to your healthcare provider? How did that feedback alter your cannabis use, if at all?)*. This non-directive, open-ended approach of qualitative interviewing encouraged participants to elaborate beyond the original scope of the interview guide and allowed for unanticipated perspectives.

We sent audio recordings of interviews to an external HIPAA-compliant transcriptionist who de-identified transcripts. The files were uploaded to a HIPAA-compliant OneDrive for team analysis. The team made summaries of emerging ideas in transcripts after each interview was completed as part of the initial analysis. As data collection continued, emerging ideas aligned with previously observed phenomena, confirming that theoretical saturation was achieved [34, 41]. This occurred once 19 participants had completed the study. We used a theoretical sampling strategy wherein data collection is completed once emerging theory aligns with previously observed phenomena and data saturation is achieved [33, 34, 39].

### Data analysis: constructivist grounded theory

We sought to develop a conceptual framework (theory or explanation) through analyses and constant comparisons across the data [33]. This methodology is best suited to exploring social processes and phenomena, with the aim of generating aforementioned theories and explanations from the data that are based on participants’ narratives and experiences [34]. Team members read each transcript and wrote initial memos based on underlying ideas emerging from the transcript. Once the final interview was completed, transcribed, summarized, and memoed, the team reviewed and categorized emerging thematic subject areas that formed the basis of a codebook. This included definitions and examples which we discussed and revised as a team (i.e., *Describing information source for cannabis use during pregnancy: learning about cannabis use for pregnancy including anecdotal evidence, where they’re getting information from*). We then uploaded the transcripts into ATLAS.ti™ data software program, Mac Version 22.1.0, and input the codebook. Then we tested one transcript from the dataset as a team and revised the codebook as needed to produce a set of 26 thematic codes for analysis (Appendix 3: Codebook).

Then, the research team split into two-person pairs wherein each team member independently analyzed a set of transcripts and pairs met weekly to discuss and construct emerging insights from their shared transcripts. Weekly analytical meetings were used to keep an audit trail, pose questions about codebook applicability, discuss observations and deviations, and co-construct theories which informed early memos [40]. This triangulation between researchers deepened understanding of theoretical concepts as we moved through stages of analysis. All members of the data analysis team met weekly with the PI to discuss coding, themes, and memos. Final memos (supervised and guided by the PI) were made to capture themes resulting from code overlap and to facilitate deeper discussions of the data at these meetings.

After developing initial findings, we conducted member checking with participants [41]. This is done to give participants additional opportunities to engage with the data and researchers, reflect on their responses to confirm or refute results, and strengthen overall findings. Member checking included survey responses (n = 7) and two focus groups with 2 or 3 participants each (n = 5) depending on participants’ response to email invitation and how they wanted to interact with the data. We provided participants with thematic categories and brief descriptions of each. Questions (i.e., *“Is this finding true to your experience?”, “What is missing from this finding?”*) were posed to participants. Then, they had the opportunity to provide open-ended responses to the categories and, in the case of focus groups, discuss their thoughts with one another on a typically taboo topic. This also allowed participants to explore the perspectives and experiences of those in similar situations to themselves and consider if additional themes related to their own experience [41]. We constructed final insights from this process of group discussions on preliminary themes to develop the three results related to changes in cannabis use in the perinatal period.

## Results

### Result 1: Participants made changes to the amount of cannabis products they used in pregnancy to reduce harm to their unborn children

Individuals reported adjusting the amount of cannabis used in a conscious effort to minimize potential harm to their unborn children. These adjustments included maintaining a minimum level of use deemed necessary for managing symptoms rather than complete cessation. Individuals expressed managing their cannabis use in this way to balance well-being for both them and their baby:

“I just continued my cannabis use [during my pregnancy]. I kept it at a low, a minimum, just in case... I still have a baby in me. And I don't want my baby being affected negatively...it was the best decision I could have made in my pregnancy. Because going without [cannabis] was just not a healthy place to be anymore.” (Participant 10)

They went on to indicate that they reduced their cannabis use during pregnancy and monitored their dosage with the help of a dispensary to a level that could effectively manage their symptoms, such as nausea and chronic pain, but not feel “high”:

“I knew that typically around 10 [MG] was the serving size that works for me. It minimizes my symptoms, and it doesn't get me to a point where I'm not cognitively all there. I'm not so much high, I'm just managing symptoms. I could still feel it, but it wasn't disorienting at all. And because I was going to a dispensary, I was able to keep it at the same level and not get too high, or under to where it's not really affecting me. [A]t the dispensary that I go to, they do really extensive testing. And they have all of their specifications listed on the products.” (Participant 10)

Some individuals explained the need to reduce their cannabis use because they felt too “high” and were concerned it would impede their ability to take care of their other children:

“I have tried the [higher doses of THC] gummies that I like… But it’s not something that’s sustainable because what happens if I need to get up and take care of my kid in the middle of the night? I don’t want to be high as a kite.” (Participant 1)

Multiple individuals reported trying to stop using cannabis when they were pregnant but feeling they needed to use cannabis again to avoid feeling sick due severe vomiting and nausea throughout pregnancy. This was often difficult because of symptoms that they were experiencing. Some participants described instances where they felt they were consuming too much cannabis and chose to abstain temporarily for their fetus’ well-being, but they felt this was often unsustainable due to the resulting physical discomfort:

“[I]f...I felt like...I'm smoking too much...I would... just have to sit and be sick...Because I did get some mom guilt... Just thinking about ...what if something happened to my baby, I would let that influence how I felt. So ... I'm not going to smoke today. I'm going to lay down. I'm going to feel this way and just go through it. But it would always change, because I would feel so physically sick that I would need to smoke cannabis.” (Participant 18)

Another participant had a similar experience where they reported using cannabis prior to pregnancy, but then attempted to stop using cannabis entirely once they learned they were pregnant to give birth to a healthy baby. Despite their desire for cessation of cannabis during pregnancy, they felt that they had to resume use to alleviate issues of pain, low appetite, and mental health:

“I thought all my life I would never ever smoke during my pregnancy. I take being a mother very serious...That’s why ...I tried not to smoke [cannabis]... [I thought I would] make some lifestyle changes, this is on me, this is my decision, I’m just gonna not smoke... when [the lifestyle changes didn’t work] my body was like no, we need to smoke.” (Participant 19)

This individual also attempted not to use cannabis at all, which was complicated by mental health symptoms they previously managed with cannabis. They noted that stigma towards cannabis use in pregnancy deterred them from what they perceived was otherwise a viable treatment modality:

“[W]hen I found out I was pregnant, I made it up in my mind that I was not going to take any cannabis at all during my pregnancy. And I was really nervous about it. I [said] I'm going to cut it cold turkey, and I'm just going to tough it out. And it didn't work out that way (LAUGHS)...I felt really negatively. My depression and my anxiety started being impacted, as well as all of this chronic pain just kind of crashing down on me. And so I decided... I need to look into this more and see if it's still an option. And just get over the fact that I didn't want to [stop using cannabis during pregnancy] purely out of the stigma.” (Participant 10)

### Result 2: Participants made changes to the types of cannabis products they used in pregnancy to prioritize options perceived as safer for a fetus

Another method individuals used to minimize potential harm to their unborn children was modulating their form of cannabis consumption. Several participants reported choosing to consume cannabis edibles instead of smoking it due to health concerns associated with smoking, especially if it was paired with tobacco as they used to consume it:

“I went from mostly smoking blunts [before pregnancy to only smoking cannabis in pregnancy] ... My midwife did let me know... smoke in general, and inhaling even vape, wasn't always ideal. I cut back a lot on smoking... and [used] edibles more [while] being pregnant…Obviously, I wasn't going to smoke tobacco while being pregnant. I knew that wouldn't be safe.” (Participant 16)

Others also noted the need to cut down on pairing tobacco with cannabis, and to be more selective of cannabis products based on length of effects. After they discussed their cannabis use with their clinician, they chose a modality which they felt would help their symptoms but not negatively impact the pregnancy:

“The only thing [healthcare clinicians] will say is no tobacco... that told me bongs, pipes, papers, the hemp wraps...teas, ...edibles [were all safe to use]... that’s six different ways off the top of my head that you can do [cannabis] naturally...it was all a matter of me finding a way that works for me... I didn’t like the way the edibles made me feel because it’s a way longer high... it lasts for up to eight hours. That’s a whole day. [LAUGHS] I don’t need that [when] I’m pregnant...I need [cannabis] for me. I don’t need my kid to be high.” (Participant 19)

Other participants avoided smoking cannabis in favor of edibles. They felt that despite the potency of edibles, they were a safer alternative that prevented exposing their developing fetus to harmful smoke:

“[T]he idea of smoking [cannabis] just felt very taboo. [It] didn’t feel like a good idea. I felt safer with the gummies, even if it maybe was... even more potent; it just felt safer [than smoking]. I just didn’t associate smoking with pregnancy at all.” (Participant 1)

Some participants reported avoiding smoking cannabis but felt confused if this was the best course of action. They shared that they wanted safer forms of ingestion, but did not have enough information to decide for themselves what a safer form might be:

“I chose to consume edibles instead of smoking because I know that smoking ... doesn't have a positive effect on pregnancy or just your health in general. That's why I decided to do edibles... while I'm pregnant... But my friend who is a nurse [said] choosing edibles has more of an effect on your body. So that's why she chose to smoke. And this is why I would like to know more about it, because I would like to choose an option ... that would have the least effect on the baby.” (Participant 4)

### Result 3: Participants made changes to where they were accessing cannabis products in pregnancy to prioritize known and trusted sources for safer consumption

Participants expressed a preference for consuming cannabis from known and trusted sources to mitigate any potential harm from consumption. They prioritized the quality and safety of the cannabis products, often choosing to use cannabis grown by family members or purchased from trusted dispensaries. One participant described how they made a conscious effort to avoid products they deemed as potentially unsafe by looking into cannabis farms and brands prior to purchasing.

“I like to know where my cannabis specifically comes from... I have growers in my family ...I just don’t go and get whatever from wherever, I like to know, and I like other people to know where they get their stuff from. And not just random [places]…I’ve used dispensaries, like when family members run out or it’s not harvest season. I do go to dispensaries a lot. I like to research the brand and the farms...I don’t like to buy blindly, just like any other medicine.” (Participant 6)

Other participants echoed the sentiment that knowing the quality of their cannabis products was important:

“We grew a bunch [of cannabis] when I was first pregnant. I was...smoking the stuff that I had grown... I know where this has come from...I know the quality of it.” (Participant 3)

Other participants emphasized that during pregnancy they were more selective about the quality and source of cannabis products they were purchasing. The participant felt that while buying cannabis from dispensaries was more expensive, it was also a way to prioritize consistent quality of the product they were buying:

“I didn’t get cheap stuff. [LAUGHS] I didn’t get a $15 cartridge or a $15 gram. It was... quality and from ... actual shops. That’s one thing that changed [when I became pregnant]. ‘Cause before [if] the homie, or so-and-so has a discount, or they made some [edibles or concentrates] ... I’ll try it, whatever. But when I was pregnant, no way. Because I didn’t know what oils they use, what chemicals were burning in there... I was very picky.” (Participant 5)

Some participants described buying their cannabis from dispensaries because they believed it provided more regulation of cannabis products. With this knowledge, they felt that the products were safer for their pregnancy than if they had bought cannabis off the street:

“I tend to stick to licensed shops. Just because everything is safer and tested. And with all these crazy stories nowadays, about people buying from people they know, and stuff having god-knows-what in it. I'm about to have two babies, I have to be very careful. So, I always strictly shop from dispensaries, either medical or recreational ones.” (Participant 9)

Participants also reported that accessing dispensaries enabled them to avoid feeling too “high” due to the regulation of cannabis products:

“[B]ecause I was going to a dispensary, I was able to keep [my cannabis use] at the same level and not get too high, or under to where it's not really affecting me. [A]t the dispensary that I go to, they do really extensive testing. And they have all of their specifications listed on the products.” (Participant 10)

## Discussion

Participants reported making changes to the way that they used cannabis during pregnancy in service of prioritizing perceived safer options for themselves and their fetuses. This included making changes to the amount, type, and source of cannabis used. Participants described being intentional with how they selected and ingested their cannabis products based on which they believed would have the least impact on the fetus while still meeting their needs for symptom management. Our data suggest that individuals are using cannabis during pregnancy in careful and considerate ways that support what they feel is responsible and safe use for themselves and their fetuses.

Evidence from our study elucidates that there is an urgent need for harm reduction information which pregnant individuals can turn to while navigating the nexus of personal and perinatal care [42, 43]. Further, equipping both patients and clinicians with evidence-based harm reduction information is a public health imperative. Harm reduction initiatives have been highly successful in context of other substance use in pregnancy and are widely regarded as the gold standard for substance use related interventions in public health [44–48]. Our findings support prior research which has identified the need for harm reduction-based data for structural interventions, health promotion, and health education among individuals using cannabis in pregnancy [28, 49].

This data is crucial to the development of evidence-based harm reduction measures during pregnancy for those who are going to continue to use cannabis, a decision which may be impacted by interpersonal, social, and structural factors 37. While complete cessation of cannabis and any substance during pregnancy is recommended by ACOG and AAP, in practice, this may not be feasible for some individuals due to various reasons, including barriers to accessing prenatal care or available options to manage pregnancy-induced symptoms [15]. Data indicates that abstinence-based messaging from clinicians can isolate patients and drive them to search for other sources of information from friends, family, or the internet, which is often anecdotal rather than evidence-based [14, 27, 50, 51]. In doing so, people experience further harm from misinformation [27, 50, 52, 53].

Research on other substances such as opioids shows that abstinence-forward and abstinence-only messaging is often paternalistic, judgmental, and stigmatizing for recipients [54]. Prior research on cannabis use in pregnancy also demonstrates that non-abstinence messaging should be a viable goal 55 as well as the importance of a harm reduction approach [18, 44, 55–57].

Pregnant individuals reported in our study that they are already informally implementing risk reduction behaviors in service of functional goals such as maintaining well-being or caring for their families. At present, little data exist on the safest modes of cannabis ingestion, especially during pregnancy [58]. Future research should examine types of cannabis products, modes of ingestion, and frequency of use for safety data to guide clinicians and their pregnant patients in making changes to their cannabis use throughout pregnancy. Additionally, understanding the safety of dispensary products, especially given the potential for inaccuracy of THC content labeling [59] is needed to address safe supply of cannabis for this population. Our findings suggest that it is imperative that the research we conduct in this field be developed via a harm reduction framework. [60, 61 The data from our study also supports prior research which points to the issue of generating stigmatizing evidence that harms the communities it claims and aspires to help [62, 63]. Further research is needed which empowers already vulnerable pregnant populations who use substances to be able to make these decisions under medical supervision and with the support of their clinician, rather than leaving them with scant reliable data at their disposal which may promote prioritization of available, unverified information instead and thus, act on misinformation or terminate care [21, 64, 65]

## Limitations

This research has limitations. First, this research was conducted in California, where cannabis is medically and recreationally legal, and thus might not be generalizable to other regions. Further work can be conducted on recreational and/ or medical availability of cannabis and its impact on mode of use decision making. Second, it might overrepresent people who are confident in their cannabis decision making. As participants were recruited via online advertisements, there was a possibility of self-selection bias. Third, data was limited to self-reported use patterns by participants before, during, and after pregnancy. Fourth, while individuals who use cannabis recreationally may vary in their needs from those who meet criteria for cannabis use disorder, addiction to cannabis was outside of the scope of this study. Cannabis use disorder prevalence has been estimated to occur in 9% to 30% of cannabis users [66–68]. We did not recruit participants who specifically had a cannabis use disorder nor did we evaluate for cannabis use disorder. The criteria for addiction or disorder were not discussed with study participants. It is beyond our scope to extrapolate cannabis use during pregnancy to disordered use or addiction as the authors have neither the position nor information to make such determinations. For those with cannabis use disorders, accessible cannabis use reduction programs are imperative. Importantly, there was no indication from our results, or the broader literature review, that addiction was a main driver of cannabis use in pregnancy [29, 69]. Instead, we primarily observed use of cannabis motivated by a desire for preexisting and pregnancy-induced symptom management [29, 69]. Fifth, we did not collect data on the use of substances outside of cannabis. The concurrent use of tobacco and cannabis has been shown to have negative neonatal health effects [70, 71]. Tobacco use alone during pregnancy also has harmful effects on the neonate [72]. Although use rates of tobacco have declined in the last two decades, more work is needed on prevention and cessation to reduce neonatal harms [73]. While other substances were not specifically screened for, tobacco was only self-reported by approximately 15% of our sample. Participants often reported that they were aware of the negative effects of tobacco (participants 16 and 19) and made changes to their cannabis use such as cutting out tobacco which they used pre-pregnancy (participant 16). Despite these limitations, this research has important implications for guiding educational interventions and research in service of reducing maternal and child health inequities. As the impacts of cannabis continue to be studied, it is imperative that pregnant individuals are not met with judgment or lack of medical care but rather are supported in their experience of caring for themselves and their pregnancy with a harm reduction approach. More data is needed on comprehensive harm reduction approaches to cannabis use during pregnancy. This requires implementation of education on these topics in healthcare settings presented by prenatal care clinicians.

## Supplementary Information

Supplementary material 1.

Supplementary material 2.

## Data Availability

Data and materials are available from the corresponding author upon reasonable request.

## Abbreviation

BIPOC: Black, indigenous, people of color
