# Supplementary material for: “I don’t need my kid to be high”: prioritizing harm reduction when using cannabis during pregnancy
Source: Harm Reduct J. 2024 Sep 9;21:166. doi: 10.1186/s12954-024-01046-2 (PMC11382473; doi:10.1186/s12954-024-01046-2)
Supplement: Supplementary file 1 — Supplementary material 1. [file 12954_2024_1046_MOESM1_ESM.docx]

**People who use cannabis during pregnancy study (IPR 2022-2023)**

**Codebook (26 codes)**

GUIDING QUESTION

When people use cannabis during pregnancy, what historic and systematic inequities do they face + where are these happening + why?

PURPOSE / INTENT

What challenges occur when a person uses cannabis during pregnancy?

**RESEARCH QUESTION**

We hypothesize that people who use cannabis during pregnancy will experience additional burdens and inequities during pregnancy and postpartum

Format:

[[**CODE NAME**]]: Definition

*Examples: data-driven instances / experiences from transcripts*

**Global / Layered codes (5)**

Provider

Family / support person

Race / ethnicity

Class / SES

Good quote

**Learning about cannabis use x pregnancy (2)**

**Describing information source for cannabis use during pregnancy:** learning about cannabis use for pregnancy; including anecdotal; where getting info from; include when source is own trial n error or proving that cannabis is ok b/c baby came out fine; trusting or not sources

*Example: podcasts about moms who use cannabis, online resources, trusting anecdotal information about cannabis vs other resources; sharing own experience of using cannabis while pregnant and working in cannabis industry; my daughter’s healthy as can be*;v *hyperemesis support group,* seeking a cannabis community or support, b/c not getting medical support; knowledge exchange that non-judgmental*; I have a cousin; she has two children and she used cannabis for her pregnancies and she has two healthy babies.”, “I had known of other people who had smoked during their pregnancies; Relying on previous experience; provider lacking info on cannabis*

**Describing accessing cannabis products during pregnancy:** explaining where and how they were able to access cannabis during pregnancy, and related challenges and stigmas; include facilitators and barriers to access

*Examples: through friends, neighbors, partner/child’s father, dispensary, experiencing acceptance from dispensary workers about using cannabis during pregnancy; figuring out safe spaces to get cannabis vs. Safety of cannabis product; accessing dispensaries for safer products despite cost; discussing personal experiences with dispensary staff regarding cannabis products used; seeking information about products from dispensaries*

**Sharing cannabis use motivations and limitations during pregnancy (4)**

**Describing cannabis use motivations for relief due to a pre-existing condition unrelated to pregnancy:** explaining why use / not use during pregnancy for physical relief of pre-existing conditions

*Examples: having a pre-existing condition like MS, Ehlers-Danlos, endometriosis, lupus*

**Describing cannabis use motivations for relief related to pregnancy:** explaining why use / not use during pregnancy for relief of pregnancy related issues; other options not working so used cannabis for pregnancy relief—last resort

*Examples: pregnancy pain, combatting weight loss, appetite, HG, sleep issues, mental health needs; post-pregnancy (wanting to use cannabis instead of pain meds after c-section)*

**Explaining motivation to use cannabis over pharma concerns during pregnancy:** choosing to use cannabis over pharmaceutical options and why

*Example: was against using Zofran; using cannabis b/c fear of pharma on baby, trying non-pharma alternatives; plant vs drug/pharma; desiring natural remedies vs conventional medicine; wanting focus beyond physical well-being*

*desiring natural remedies vs conventional medicine; wanting focus beyond physical well-being; wanting natural*

**Receiving support for cannabis use during pregnancy:** gaining support when deciding to use cannabis during pregnancy; Experiencing support from social circle, ability for frank discussion; support people’s concerns for baby consequences. Family is supportive of it in pregnancy.

*Examples: partner supporting decision to use cannabis, parents supporting decision to use cannabis, friends/other community members providing access to cannabis, other pregnant people in social circle who used cannabis, comparing personal use to that of peers, social support for using cannabis in place of other substances;* providers suggesting monitoring cannabis use during pregnancy; *continuing use but discussing with provider at appointments, provider suggesting patient use/continue to use cannabis*

**Changing cannabis use patterns during pregnancy (7)**

**Using cannabis to feel normal (feel good, good choice):** using cannabis for marginal benefits/ to enable being a good parent, carry out regular duties

*Example*: *without cannabis I don’t think I would have even gotten through;it's not like that cannabis was like a million times better than the medicine [14]; give me like the strength to just be okay and enjoy a family movie night with my family [1]; I feel like they think you’re just getting high just to be it, you know. Just to feel high but honestly it makes me feel normal.*

**Feeling internal conflict about cannabis use during pregnancy:** perceptions of how it would look using cannabis during pregnancy, feeling bad about using

*Example: Feeling internal struggle to use b/c felt wrong during preg; feeling “mom guilt” to smoke less during pregnancy*

**Aiming for responsible and safer cannabis use and patterns during pregnancy:** balancing desire for cannabis benefits with need to be a mom by changing cannabis use patterns

*Example: choosing weaker cannabis options to maintain capabilities as a parent; I need to get up and take care of my kid in the middle of the night? I don’t want to be high as a kite, you know. [1]; changing cannabis use patterns to alleviate concerns over baby development; aiming for responsible cannabis use, balancing desire for drug benefits with need to be a mom; balancing individual and family needs; heavy cannabis user before pregnancy; stopping to keep family happy; creating smoking routine to cut back, I did stop recently because my baby’s now starting to roll and he’s starting to play with things [1]; stopping or avoiding altogether cannabis; I feel like I’m a little cleaner, you know, as far as like letting it [ventilize?] [5]; Use discreetly, so not smell—not want people to know; I just didn’t associate smoking with pregnancy at all. [1]*

**Comparing cannabis vs. other substances:** describing cannabis-substance trajectories or harm reduction reasoning comparing cannabis to other substances

*Example: meth*

**Reflecting on tobacco-cannabis use during pregnancy:** describing cannabis-tobacco trajectories or harm reduction reasoning comparing cannabis to tobacco/nicotine/vaping products

*Example: it’s really crazy to me that people can like smoke cigarettes, there’s so many other things you can do during pregnancy that should have more backlash... it’s natural, it’s grown from the earth. [6];*  *discontinuing tobacco co-use once pregnant, using cannabis with meth, using cannabis with/without tobacco during pregnancy*

**Using cannabis during lactation:** decision making around how to breastfeed while consuming cannabis

*Examples: continuing cannabis use, not receiving info about cannabis use and lactation, avoiding during nursing; needing to do own research on if safe during nursing; receiving conflicting info on breastfeeding and cannabis; avoiding during nursing,*

**Using cannabis to get high:** Using cannabis beyond symptoms relief, seeking high feeling

*Example*: *I just like to be high [5]*

**Dealing with health system and providers while using cannabis during pregnancy (8)**

**Fearing CPS involvement for use of cannabis during pregnancy:** worrying about CPS involvement for using cannabis during pregnancy

*Examples: hearing other people’s experiences with CPS due to cannabis use, past personal experience/contact with CPS, doctors threatening to involve CPS*

**Distrusting individual provider and their care b/c they are an “user” (interpersonal):** Experiencing discrepancy between personal experience vs. provider comments; pt describing feeling not heard by provider [vs. Variation in care codes, consequence of not being heard]

*Examples: not feeling heard; felt like I always had to keep repeating myself, it was just twice; feeling distrusted; hiding severity of symptoms from provider, experiencing disbelief from HCP as a result; “try to fight it and not look crazy, throwing up every time I would sit down or stand up. But she just didn’t believe it.”; feeling a lack of transparency from health provider, causing distrust and neglecting health info*

**Getting variations in pregnancy care b/c I use cannabis:** getting diff or worse care, diff standards of care b/c of cannabis use

*Examples: provider not providing quality care, stopping care, being short with them on responses bc they use cannabis; provider care changed once tested positive for cannabis use, provider just saying “no” without providing reasoning or resources*

**Getting variations in care, tx, b/c of who I am [not connected to cannabis]:** being treated differently by provider b/c of who they are, such as SES, race/ethnicity [can double code with global codes]

*Examples: “I would say partly being, you know a woman. And then with the double impact of being a person of color, you just don’t get believed. Like people don’t believe you.” thinking patient-provider background differences prompted assumptions*

**Describing process of sharing (or not) cannabis use to provider:** walking thru moments of telling or not telling provider of their cannabis use; disclosing use or not and why

*Examples: fear of stigma due to disclosure, never asked screening questions, refusing to test*

**Shifting how I talk about cannabis now b/c of past pregnancy:** changing how I talk or use cannabis based on past pregnancy

*Examples: Having previous bad experience with health system-->color how disclosure, mistrust for subsequent pregnancies; Not disclosing cannabis use b/c of previous fears with other state legalization, conseq*

**Experiencing judgement and stigma for cannabis use during pregnancy:** pt perspective of how provider views them for using cannabis during pregnancy

*Examples: feeling shamed by providers when disclosing cannabis use, they shamed me—made me feel bad for using cannabis*

**Tracking, metrics and surveillance bc use of cannabis during pregnancy:** tracking of medical records, urine testing, and drug testing of baby b/c of cannabis use

*Examples: feeling irritation of frequency of urine testing without proper explanation to why, omitting letting know*

*Fearing disclosure of cannabis use following pt throughout care - similar to other instances in health record*

*I smoked a cigarette, like I tried a cigarette once. And then that stayed on my health record until I got older [5]*

*Mistrust, surveillance of preg b/c use of canna;like what are you even testing for with the pee? [1]; Experiencing surprise drug testing for baby postpartum*
