# Supplementary material for: “I don’t need my kid to be high”: prioritizing harm reduction when using cannabis during pregnancy
Source: Harm Reduct J. 2024 Sep 9;21:166. doi: 10.1186/s12954-024-01046-2 (PMC11382473; doi:10.1186/s12954-024-01046-2)
Supplement: Supplementary file 2 — Supplementary material 2. [file 12954_2024_1046_MOESM2_ESM.docx]

**SETUP**

- Our discussion will be about 45-60 minutes.
- E-Consent: We will be recording audio and video for documentation purposes via Zoom + confidential + de-identified.
- We may interrupt you to be mindful of your time and to get to all of our questions we would appreciate your opinions on.
- My colleagues are here as well + may jump in or have some follow-up questions for you in last 15 minutes of our discussion.
- There are no right / wrong answers; we just want to get your perspective. Assume I’m naïve!
- How data used: We will talk to 15 others + record, accuracy, share w/team + importance of contribution + compensation
- Positionality: Lead/co-lead + pronouns + positionality / privilege / social positions

**GUIDING QUESTION**

- When people use cannabis during pregnancy, what historic and systematic inequities do they face + where are these happening + why?

**PURPOSE / INTENT**

- What challenges occur when a person uses cannabis during pregnancy?

**RESEARCH QUESTION**

- We hypothesize that people who use cannabis during pregnancy will experience additional burdens and inequities during pregnancy and postpartum

| **LEVEL OF INFLUENCE** | **FACTORS / CONTEXT** | **INTERVIEW QUESTIONS** |
| --- | --- | --- |
| Individual | **Reasons for use**,  Beliefs, coping mechanisms  **Access**  Insurance coverage  Healthy literacy  Educational level  Socioeconomic status  Income | How did you learn about cannabis for pregnancy? (resources, info, dispensary)  What info did you wish you have? What’s missing?  Are there resources you wished you had access to regarding cannabis use during pregnancy?   - Do you ever feel like some resources aren’t for you? - Barriers to these resources? (income, insurance, locating resources) - Are there resources you wished you had during pregnancy?   What support would you like to see around cannabis for pregnant people? (probe: policies, laws, for parents, for nursing) |
| Interpersonal | **Cannabis visibility, acceptance**  **Cannabis culture**  Family, Parents  Friends, Peers  Teachers, Educators, Supporters | Who do you talk to about your cannabis use, if anyone? (Probe: nurse, social worker, health care provider; family / partner / friends, OB-GYN)   - What kind of feedback did you receive? - How did that feedback alter your cannabis use, if at all? - How normalized was cannabis use growing up? - How is cannabis viewed within your family? friends? partner / spouse? (growing up-->now during pregnancy) - Who did you use cannabis with before/during your pregnancy? - How is cannabis use during pregnancy viewed by people supporting you with the pregnancy? (family, friends, partner/spouse) - What feedback did you get from family and friends about your use of cannabis during pregnancy? - Did you feel comfortable letting providers know about using cannabis during pregnancy? (prenatal care, during birth, social worker...) - What reasons did you share with your provider? - What kind of feedback did you receive from your provider? - How did that feedback alter your care? (Probe: visits, trust, quality of care, switch providers or institutions?) - Were there any specific factors / reasons for using those particular products / type of cannabis? - How easy / hard is it to get cannabis where you are? What barriers are there to getting the cannabis you want? |
| Community | **Care access / quality / type**  Low income, less options  Ability to change doctors  Less frequent visits  Substance use tx availability, access  **Generalized support**  Info for using during pregnancy  Substance use disorder tx  Resources at prenatal care site, elsewhere  **Cannabis visibility/ acceptance in community**  Shop prevalence/ types (boutique vs bunker) | - Where do you get health info about pregnancy? (info sources about pregnancy, cannabis x pregnancy) - Where do you get healthcare info about pregnancy? - How did you choose where to get healthcare info about pregnancy? (engaged in prenatal care, how choose provider / institution, where else getting pregnancy care?) - What was access to healthcare like during your pregnancy? - Prenatal-nursing-postpartum - How did you choose to feed your baby, and what factors went into that decision?   - (if decided to nurse) Lactation support: What were your options around breastfeeding / nursing with cannabis? - Mental health / wellness / therapy - In-person / online care, community support groups, etc. - How often you could access, near / far - What was the care team like? (different roles – OBGYN, social worker, nurse, mental health provider, community leader, etc.)   - What were their feelings towards cannabis use? (institutional vibe beyond policies)   - Fave / least fave care team member   - Did your care team look like you? (probe: welcoming, affirming space vs. medical mistrust, address historical trauma, assumptions, treated differently b/c of who you are + cannabis) - How did you feel about the quality of services provided by X?   - What was lacking / helpful? (how competent/ complete – non-medication resources not necessarily offered at low-income clinic sites)   - What services / resources / info did they have about cannabis during pregnancy? (probe: substance use disorder treatment, support groups, competent education, etc.)   - What resources / support would’ve you liked to have had?   - Could design your own pregnancy experience for yourself or a girlfriend who uses cannabis, what would you want to see? What would you want to be different? (probe: postpartum, nursing) - Where did you purchase/ use cannabis? (probe: safety, recs of products to use during pregnancy, administration + mode; how changed across pregnancy, trust)   - Local shops, online, corner shop, trades   - Ability to use on-site – community get-togethers   - Preferred products / admin, and when? (early, late, birth, nursing)   - What products do you prefer to use / not use during pregnancy + postpartum? (probe: tapering) |
| Societal | **Cannabis laws and policies**  Prenatal care guidelines  Cannabis and substance use screening during prenatal  **Negative consequences**  Criminalization and child services  Discrimination and stigma  **Social norms**  Social media and ads | - Can you tell me about the times that providers talked to you about cannabis use since you found out you were pregnant? - Can you tell me about the times you had to provide an urine test? (told in advance, done b/c disclosed cannabis, threats of child services, every visit) - Who did you have conversations with about using cannabis during pregnancy? (providers, stigma, discrimination; disclose vs not) - Do you feel your care changed b/c of disclosing cannabis use?   - Relationship with particular provider / health system? (probe: changed providers, health systems—why this health system?--more open to cannabis)   What were some positive / negative experiences you had related to using cannabis during pregnancy?   - Probe: provider / medical system related - Probe: criminalization, child services, threats with justice system, discrimination and stigma   Can you describe to me any experience you had where you felt you were treated differently after someone found out that you used cannabis while pregnant?   - If you were X [white, different health system, insurance...immigration status, etc.], do you feel your experience with cannabis and provider / health system would have been different? - What do you wish you could tell your provider about people who use cannabis during pregnancy—what do you feel they get wrong about you?   Where do you get info about cannabis x pregnancy? (social media, friends, budtenders—have them name it)   - What sources do you trust to receive health information? - How common or uncommon do you feel cannabis use during pregnancy is (among their friends / peers)? |
